# Perceived discomfort and neuromuscular fatigue during long-duration real driving with different car seats

**DOI:** 10.1371/journal.pone.0278131

**Published:** 2022-12-12

**Authors:** Mathieu Lecocq, Pascaline Lantoine, Clément Bougard, Jean-Marc Allègre, Laurent Bauvineau, Damián González, Christophe Bourdin, Tanguy Marqueste, Erick Dousset

**Affiliations:** 1 CTAG, Centro Tecnolóxico de Automoción de Galicia, Porriño, Spain; 2 CNRS, ISM, Institut des Sciences du Mouvement: Etienne-Jules MAREY (UMR 7287), Faculté des Sciences du Sport, Aix-Marseille Université, Marseille, France; 3 Groupe Stellantis, Centre Technique de Vélizy, Vélizy-Villacoublay, Cedex, France; University of Hartford College of Education Nursing and Health Professions, UNITED STATES

## Abstract

**Introduction:**

Identification of the seat features that could improve driving experience is a main issue for automotive companies.

**Objective:**

Long duration real driving sessions were performed to assess the effect of three seats (soft–S_1_, firm–S_2_ and suspended–S_3_) on perceived discomfort and neuromuscular fatigue (NMF).

**Materials & methods:**

For each seat, the muscular activity of bilateral Trapezius Descendens (TD), Erector Spinae (ES) and Multifidus (MF) muscles of twenty-one participants was recorded during real driving sessions of 3-hours each lasting approximately 3 hours and following the same itinerary. During each driving session, participants were also regularly asked to self-evaluate their level of whole-body and local discomfort. In addition, an endurance static test (EST) was performed before (ESTpre) and after (ESTpost) each driving session to assess the seat effect on physical capacity.

**Results:**

Whole-body discomfort increased with driving time for all seats, but this increase became significant latter for S_3_. The highest scores of local discomfort occurred for neck and lower back. Contrary to S_1_ and S_2_, the duration of ESTpost was not significantly lower compared to ESTpre with the S_3_. Interestingly, muscular activity of S_1_ remained stable throughout the driving task which could be attributed to sustained muscular contraction, while muscular recruitment adjustments occurred for S_2_ and S_3_ from 1H00 of driving. This muscular compensation concerns mostly the right side for S_2_ and S_3_ but with different profiles. On the left side, the muscular adjustments concern only the MF with S_2_ and the ES with S_3_.

**Conclusion:**

Overall, our results demonstrated that S_3_ could be considered as the most suitable seat to delay discomfort and NMF appearance.

## Introduction

Driver fatigue is questioned to be a main source of car accidents [1]. Such fatigue could be related to many factors such as sleep deprivation, road monotony, constancy of the sound environment [2, 3]. Moreover, perceptual-motor adjustments involved in driving task (i.e., actioning the pedals, using the steering wheel, select trajectory, traffic management) are also potential factors contributing to fatigue [4]. Thus, it could be considered that fatigue induced by driving task is related to multiple interactions between environmental (weather, cars traffic, road types), psychological (mood, mental workload) [5] but also physiological factors (such as muscle activity, force production, postural regulation, pain…) [6]. Consequently, neuromuscular fatigue should also be taken into consideration regarding the effect of prolonged driving position [7, 8]. Maintaining a stable posture during driving induces a decrease of blood flow in local muscles [9] and within intervertebral disks [10]. According to several studies [11–13], unchanged position emphasizes neck, shoulders and lumbar pain. Bongers and Boshuizen [11] have demonstrated that these symptoms could also be induced by seat vibrations. The latter associated to decrease in local blood flow leads to postural adjustments approximately every 5 minutes [14]. Winkel and Westgaard [15] and Kruizinga et al., [16] have shown that, during driving task, postural changes mainly implicate muscles involved in neck maintenance, trunk stabilization and upper/lower limbs movements. However, these postural changes are limited because of the driving task constraints. Consequently, the fixity of the position induces the recruitment of the same muscles during the driving task which, with time, could lead to reduction of local (muscles and intervertebral disks) blood flow [9] and intervertebral disc nutrition [17, 18]. Such processes associated with neuromuscular fatigue may also induce increase in perceived discomfort [8, 19].

These processes are in accordance with the multidimensional characteristics of NMF. It is now commonly admitted that NMF originates from four levels, i.e., task dependency, muscle capacity to maintain a task, neural strategy to prolong the task and perception of effort related or not to motivation [20, 21]. Regarding such complexity, NMF could be defined as different task-dependent profiles characterized by specific process of adaptation to the task. Considering this, it is not surprising to observe that prolonged driving task represents a high risk of lower back pain which in turn leads to neuromuscular fatigue [22].

Neuromuscular fatigue in the automotive context is characterized by an increase in temporal variability in EMG amplitude of the back muscles and trapezius muscles [23, 24]. However, the nature and number of studied muscles, seats tested, driving conditions (real or virtual environment, with or without vibrations) and driving time represent a great source of variability in experimental protocols. For instance, Hostens & Ramons [13] asked their participants to drive on a chosen road which contained only left turns during 1 hour. The angle of seat pan and backrest was set at 110° for each participant. They reported that half of their subjects perceived trapezius and deltoid stiffness following the driving task. In their study, El Falou et al., [8] evaluated muscular activity and perceived discomfort for two different seats with and without an applied vibration. They found that discomfort level increased throughout the 150 minutes for both condition while no significant modifications of muscular activity were observed in cervical erector spinae and oblique muscles. In another study where participants performed six driving sessions each of 15 minutes on a static simulator, Maradei et al. [25] showed that the erector spinae muscle activity increased during the first 45 minutes and then decreased. In similar conditions, Balasubramanian et al., [26] reported an increase of neck and shoulders muscular activity but they considered this period to be too short to be related to neuromuscular fatigue. Gyi and Porter [27] suggested that a minimum of 2 hours of driving are required to clearly assess seat comfort and any subsequent neuromuscular fatigue. Sustained contraction which is commonly observed in driving tasks induced an increase in discomfort levels and neuromuscular fatigue [28, 29]. De Looze et al., [30] suggested that a too low muscular activity could foster too much comfort leading to drowsiness. In this context, car seats appeared to be a key factor in maintaining muscle activity within an optimal range to ensure security, effectiveness while still providing an acceptable level of comfort. Indeed, an inappropriate seat design could result in unsafe mental and physical workload during driving [31]. A too long cushion could increase thighs pressure inducing local discomfort and legs blood flow reduction [32]. Similarly, Na et al., [33] demonstrated that a poor positioning relative to seat’s support leads to lumbar misalignment associated with difficulty in reaching the pedals, blood flow reduction and exhaustion from constant legs strain to support chosen driving position. So considered pressure distribution, muscles activity and spinal column curvatures are key factors in reducing driver fatigue [30]. Depending on their shape and on their constitution (more or less firm), car seats could modify the body maintenance and therefore the muscular activity necessary to maintain the driving position.

In a previous experiment [34], we assessed the effect of two different seats (one soft, one firm) on neuromuscular fatigue during long driving duration on a static simulator. Our results demonstrated that for both seats neuromuscular fatigue occurred with specific seat-dependent profiles. The softer seat induced higher muscular activity of lower back muscles with contralateral compensation, while the firmer seat offered greater support for the lower back. However, participants did not consciously perceive a difference in neuromuscular fatigue levels between seats as the self-perception of body discomfort indices were not significantly different from one seat to another [34]. The merit of our study was to objectify the effect of seats on neuromuscular fatigue demonstrating a potential interest in firmer seats. The latter seems more adapted to promoting compensation strategies through its firmer support. However, this study was realized in static simulated conditions. We have to question the immersion of subjects due to the absence of real driving conditions (i.e., real risks taken as well as accelerations, reaction forces and vibrations stimuli). For these reasons, the present study aimed to assess neuromuscular fatigue in real driving conditions according to the firmness of both seats already tested in our previous study but also the addition of a new seat prototype which is a suspended seat (containing a new technology developed by the Stellantis group). This technology comprises a short vibration absorber (mechanical spring) directly included in the seat backrest. The main goal was to assess how the type of seats (firm, soft, suspended) but also real driving conditions could affect the level of neuromuscular fatigue during long duration (3 hours) driving. We hypothesized that the suspended technology could reduce neuromuscular fatigue and perceived discomfort by decreasing the level of whole-body vibration (WBV) transmitted to the drivers.

## Materials & methods

### Ethical approval

The present study was approved by automotive manufacturer Stellantis’ “Comité d’hygiène, de sécurité et des conditions de travail” (Hygiene, Safety and Working Conditions Committee) and the research site was validated as biomedical research site n˚15–225 by the “Agence Régionale de Santé—Ile de France” (regional health agency Ile de France). Moreover, ethic approval was given by the ethics committee of CERSTAPS (CNU–IRB000112476-112).

### Subjects

Twenty-one participants with a mean age, height and weight of 27.8 ±5.6 years, 1.7 ±0.1 meters 74 ±13.7 kg respectively, volunteered to take part in this study and to perform at least two sessions. Each participant was naïve regarding the aim of our experiment and the type of seat tested. The inclusion criteria was to not have relevant signs of musculoskeletal trouble or back pain, to not be pregnant and presented at least two years of driving experience. Before each experimental session, participants were informed about the purpose of the task to be carried out during the experiment and gave their written informed consent (as outlined in PLOS consent form).

### Seats features

The physical description of the three seats tested in our experiment as well as their softness characterization tests performed by CTAG (Automotive Technology Center of Galicia, Spain),) are already well detailed in the article of Lantoine et al., [35]. These tests consisted of measuring the maximum displacements of cushion and backrest foams under an identical force applied by a robotic dummy (Force vs Displacement tests—FvsD). Briefly, the results demonstrated that FvsD for the backrest was nearly similar for the three seats. Concerning cushions, the foam displacement was highest for S_3_ (suspended seat), followed by S_1_ and finally S_2_, thereby defining S_3_ and S_1_ as soft seats and S_2_ as a firm seat. For more details, see Lantoine et al., [35]. It is important to notice that due to confidential and patented data, we are unable to provide more details regarding seat features.

### Experimental synopsis

To overcome the effect of circadian rhythm between subjects [1], each experiment started at 7:30 am. Firstly, each participant was asked to wear shorts and a t-shirt sportswear provided by the experimenters (same for all participants). This eliminated the effect of clothing on perceived discomfort. Following EMG electrodes placement, EMG signals were tested for each muscle of interest. Participants then performed the first endurance static test (ESTpre) to assess their initial physical capacity (i.e., before the driving session). Then, participants were asked to sit in the vehicle cockpit and to adjust themself the car seat to feel as comfortable as possible according to French driving rules. Following their installation, each participant was asked to maintain their hands on the steering wheel, their right foot on the throttle pedal and to stay in this position for a short period (2 min) during which EMG signals were recorded. This EMG signal corresponds to the basal muscular activity, which is only related to seat settings and driver’s position and was used as a means of calibration. The participants drove a short distance (250 meters) to reach the starting point of the route. Here, the last instructions were given to the drivers and when they indicated they were ready to start the driving task, the acquisition systems were launched synchronously. At this point, each participant started to drive following the same itinerary displayed on a GPS system. Two experimenters were present inside the vehicle to ensure the smooth running of the experiment. Finally, at the end of the driving session, the participants performed the endurance static test a second time (ESTpost).

### Vehicle and driving session

**Fig 1:**
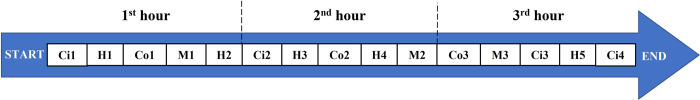
Chronologic order of road sectors throughout the driving itinerary. Each participant followed the same itinerary, which included city (Ci), highway (H), country (Co) and mountain (M) roads.

For this experiment, driving sessions were performed in a 3008 Peugeot (puretech 130) with an automatic gearbox (EAT6). Some pre-tests were made to define an itinerary requiring at least three hours of driving, not subject to traffic jams and containing city (Ci), highway (H), country (Co) and mountain/sinuous (M) roads. A programmed GPS allowed each participant to follow this itinerary. Chronological order of the sectors was organized as shown in **Fig 1**. Participants had to drive in the most natural way possible, respecting the French speed limits and usual driving rules. The gearbox was automatic, so participants had to only use their right foot to interact with gas and brake pedals. Moreover, they were asked to keep their hands on the steering wheel at a common 9 and 3 position throughout the driving session.

### sEMG placement

In order to assess the effect of prolonged driving on neuromuscular fatigue, the neuromuscular activity of six different muscles were recorded using a bipolar sEMG system (Biopac®, Cerom, France). These muscles were right and left Trapezius descendens (TD); which are involved for the stabilization of the head and the movements of the arms; Erector Spinae Longissimus (ESL) and Multifidus (MF); which ensure the maintenance of the spinal column. For all these muscles, the surface of the skin was shaved if necessary and cleaned with an alcohol swab to kept resistance below 5 kΩ. Then, EMG electrodes (Ag/AgCl-Electrodes Universal, Controle Graphique Medical, France) were positioned according to international recommendations [36], i.e., spaced of 2 cm apart with a recording diameter of 10 mm and fixed with adhesive plaster. For TD, electrodes were placed at the middle of the line from the acromion to the spine on vertebra C7 in the direction of the line formed by these two anatomical elements. For the ESL, the electrodes were positioned at 3 cm width lateral from the spinous process of L1 vertebra and in a vertical direction. For MF, electrodes were aligned with a line from caudal tip posterior spina iliac superior to the interspace between L1 and L2 at the level of proc. spin. L5. Reference electrodes were placed on the acromion for TD and on the superior part of iliac crest for ESL and MF. In order to assess repeatability of sensor placements over multiple sessions, electrodes placement was carefully marked on the skin.

### Endurance static test

**Fig 2:**
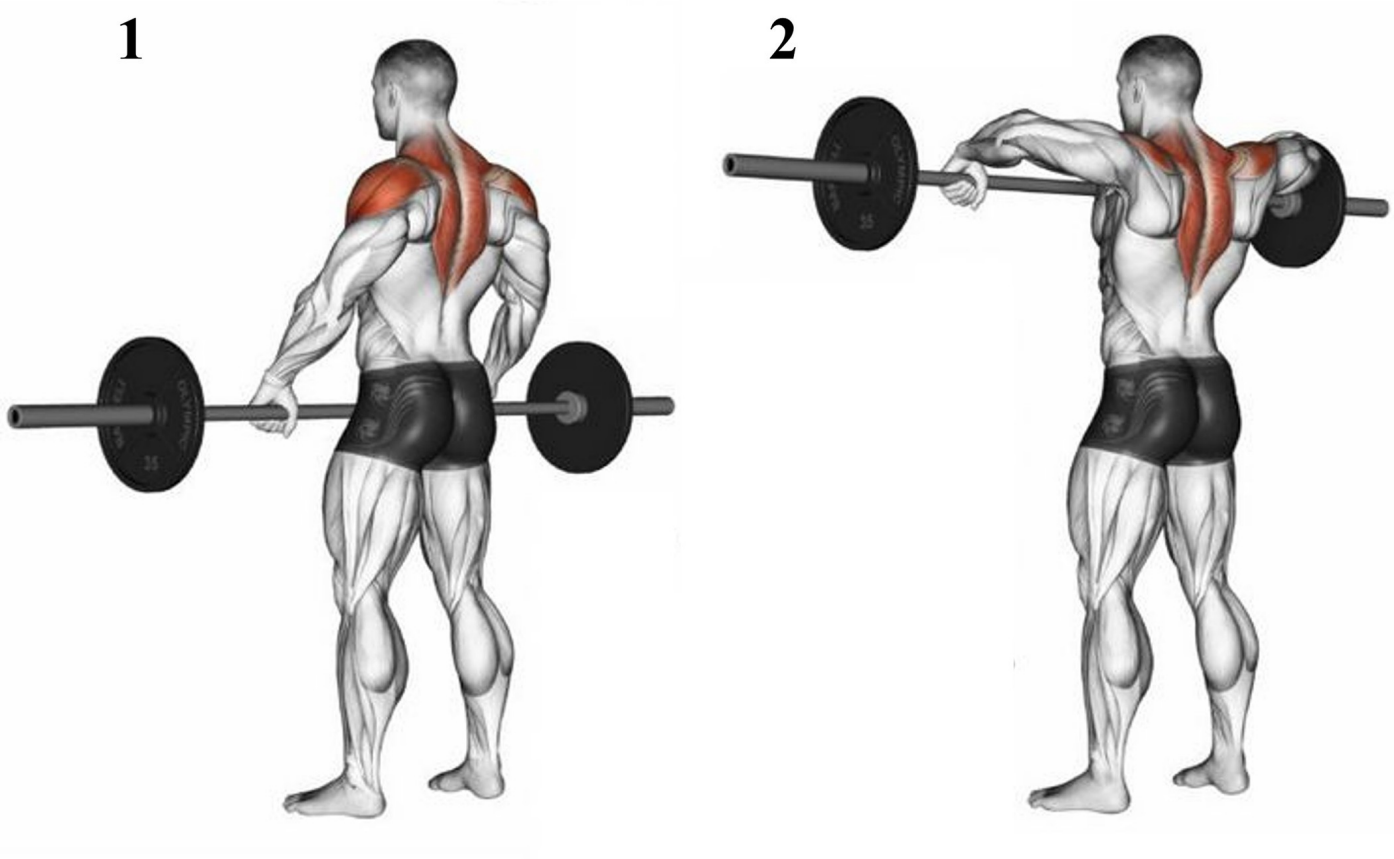
Illustration of endurance static test performed before (ESTpre) and after (ESTpost) the driving session.

Participants performed an endurance static test before (ESTpre) and after (ESTpost) the driving task. This test consisted in maintaining a weight bar at pectoral level as long as possible, as described in **Fig 2**. The load of the weight bar was adapted to the physical capacity of each participant and was unchanged for both EST. The measurement of the endurance time for both EST allows assessing the effect of driving session on TD physical capacity.

### Neuromuscular activity during driving

Once the participants have reached the starting point of the itinerary, the acquisition systems (Biopac®, Cerom, France) were launched then a new sEMG recording was performed synchronously with the beginning of the driving task and acquired at a sample rate set to 2 kHz. Concerning data analysis, signals were amplified (x10,000) and raw signals were band passed filtered from 10 to 500 Hz by a Butterworth 2nd order filter. Signals were then rebased to mean, rectified and RMS-EMG calculations were performed. As the sEMG amplitude could vary significantly between muscles, participants and recording sessions EMG signals were normalized so that comparisons between muscles and participants can be performed (Aaras et al., 1996). In the present study, considering that MVC of studied muscles is very difficult to assess with accuracy, our normalization procedure consisted in analyzing sEMG data with respect to the corresponding RMS recorded during calibration period (i.e., when participants stayed static during two minutes with the hands on the steering-wheel). Moreover, to minimize source of variability in the signals, we paid careful attention to maintain stable temperature as electrical environment within the car during all sessions as well as to electrodes placement and fixation. Moreover, participants were asked to wear identical sportswear (shorts, and T-shirt), provided by the experimenters to avoid any clothing effect. Finally, to take into account that the initial recordings mainly reflect the subject’s preparation to perform the task, all RMS-EMG values were expressed as a proportion of the the first RMS data of the driving task (i.e., the RMS recorded during the first 10% of time of the first road sector).

### Discomfort perception

During the driving session, participants were regularly asked to self-evaluate their level of whole-body discomfort on a visual analogue scale. This device was such that, the side shown to the participants included only one slider cursor while on the other, reverse side, visible only to the experimenters, the scale was graduated. Every twenty minutes, participants adjusted themselves the position of the cursor and the corresponding numerical score was recorded by the experimenter. Following this evaluation, participants were also asked to verbally give a discomfort score for each body part between 0 and 100, where 0 represented an absence of discomfort and 100 represented the maximum discomfort they could imagine. The choice to split this evaluation for each body part was made to identify where discomfort was the most elevated according to time and to car seats.

### Statistical analysis

Data were expressed as the mean ± standard deviation. All statistical analysis were computed with STATISTICA^®^ software (version 6.0). Results of EST were compared using a paired t-test (comparison before vs after driving task for each tested seat) of normalized RMS values. A two-way ANOVA (3 seats x 10 discomfort scores) was performed to compare whole-body discomfort data. More precisely, comparison analysis was done: (i) according to the initial score of whole-body for each seat (time effect) and (ii) at each time of assessment, the whole-body discomfort scores of all seats were compared (seat effect). When significant difference was observed, statistical analysis was completed by a Tukey-Kramer post-hoc. The same process was performed to analyze the evolution of discomfort level for each body area. To compare the effects of car seats and road sectors on neuromuscular activity, normalized RMS values of each muscle were compared using a two-way ANOVA (3 seats x 15 road sectors). A Bonferonni post-hoc test was applied when a significant effect was observed. All statistical differences were considered significant when p<0.05.

## Result

### Endurance static test

**Fig 3:**
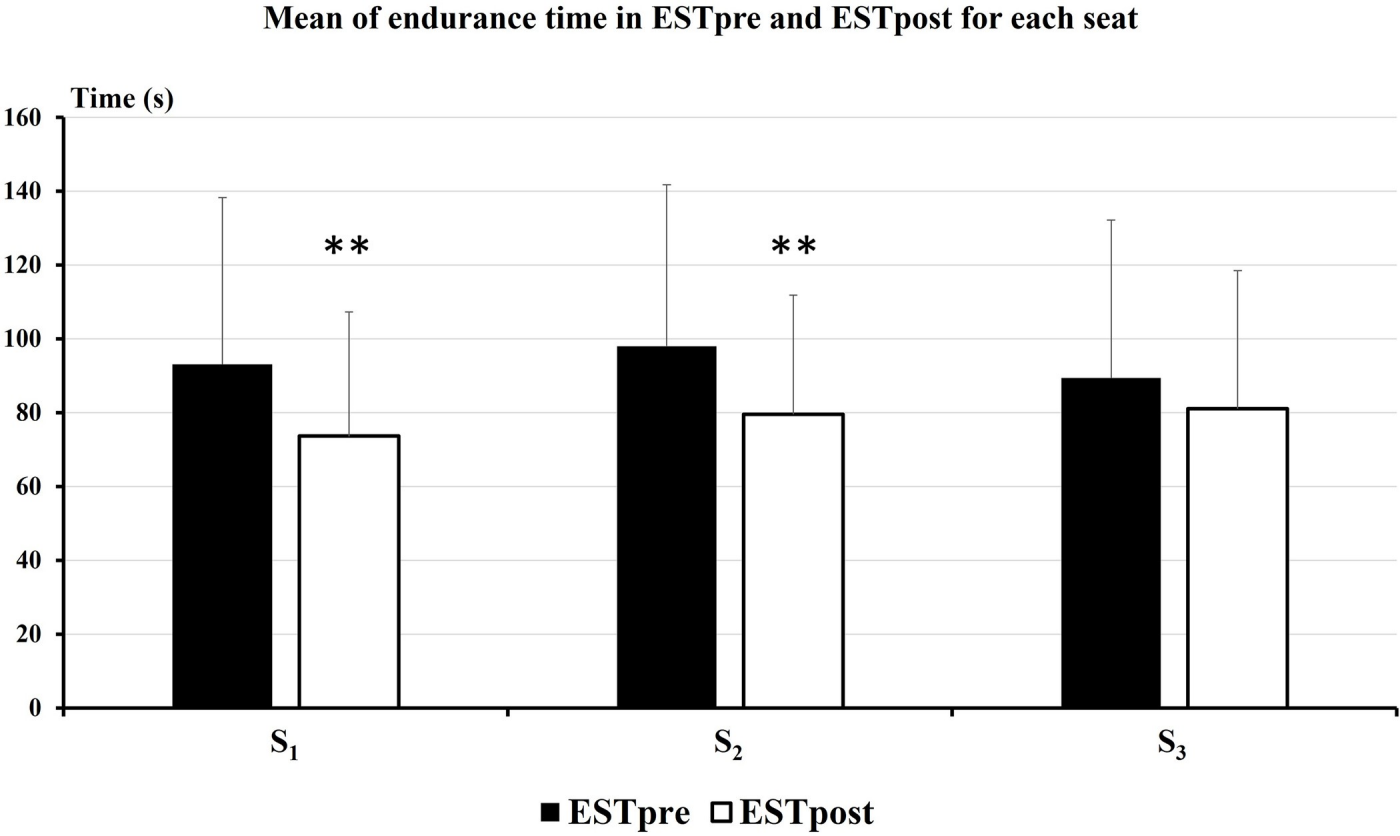
Endurance time mean (in seconds) of ESTpre and ESTpost driving session for the three tested seats. Significant differences between endurance time measured before and after driving session are illustrated by * (p<0.001).

Endurance time of ESTpost was significantly shorter in comparison with ESTpre for both S_1_ and S_2_ seats (p<0.01 for S and p<0.05 for F) (**Fig 3**). However, the endurance time after driving session was not significantly different compared to before for S_3_. The endurance time was on average 19 seconds shorter after, compared to before driving session for S_1_ and S_2_ seats and only 8 seconds shorter for S_3_.

### Discomfort level

**Fig 4:**
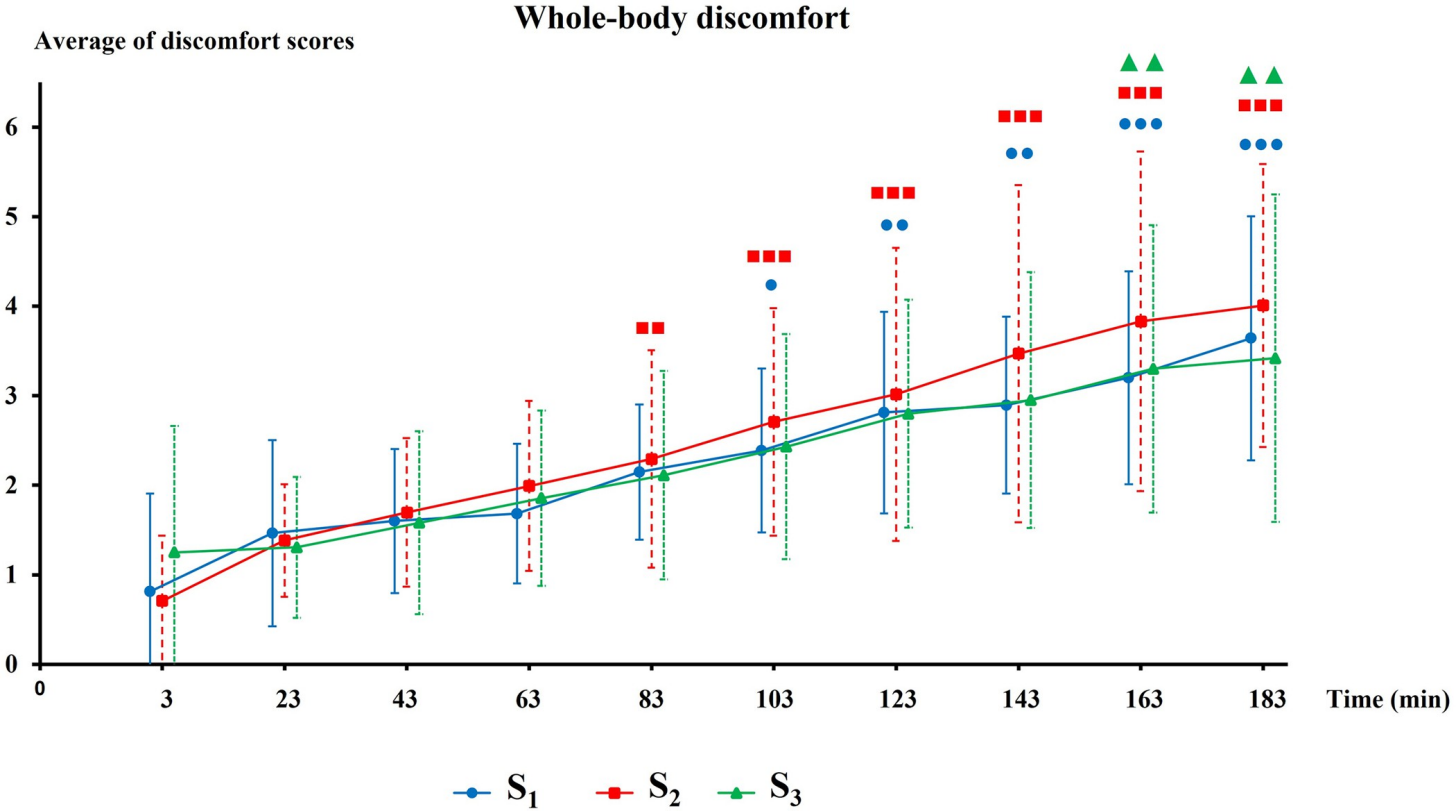
Evolution of whole-body discomfort during driving session. Significant level of discomfort compared to the initial value are represented by ● for S_1_; ■ for S_2_ and ▲ for S_3_ (1 symbol; p<0,05; 2 symbols: p<0,01 & three symbols: p<0,001).

**Fig 5:**
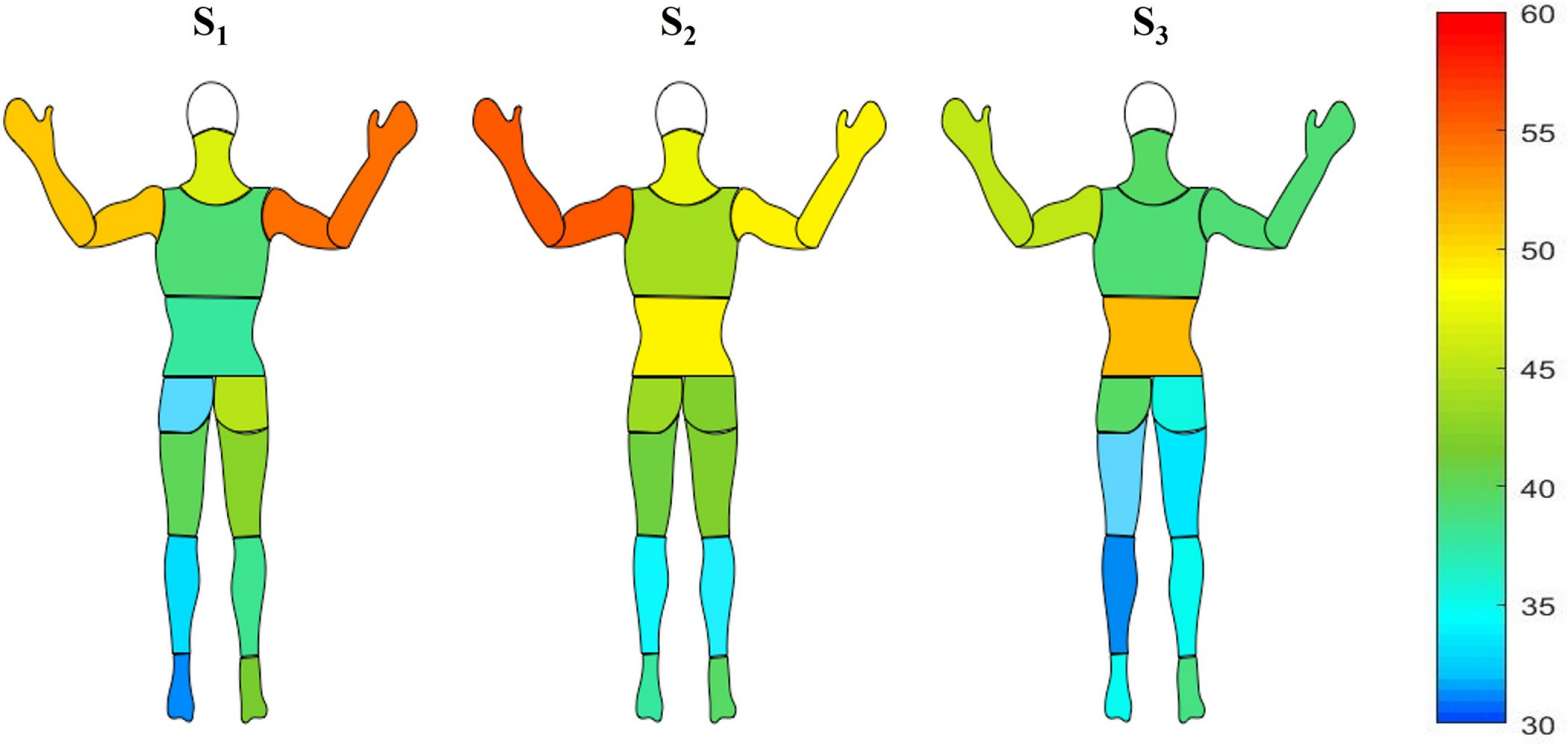
Local discomfort scores according to each body part for each seat.

For all seats, the level of whole-body discomfort increased continuously until the end of the driving session (**Fig 4**). However, this increase started to be significant from 80 minutes for S_2_ seat and from 100 minutes for S_1_ seat while it was significant solely from 160 minutes for S_3_ seat. Nevertheless, the level of whole-body discomfort was not significantly different between the three seats throughout the driving session. **Fig 5** represents the body mapping of discomfort score for each body part and for all three seats. The body areas the most affected by discomfort is the neck for S_1_; the neck and the lower back for S_2_ and the lower back for S_3_ seat. The discomfort level of the arms was also quite elevated for S_1_ and S_2_ but not for S_3_: The discomfort level of hind limb remained very low for all seats but seemed to be lower with S_3_.

### Neuromuscular activity during driving

**Fig 6:**
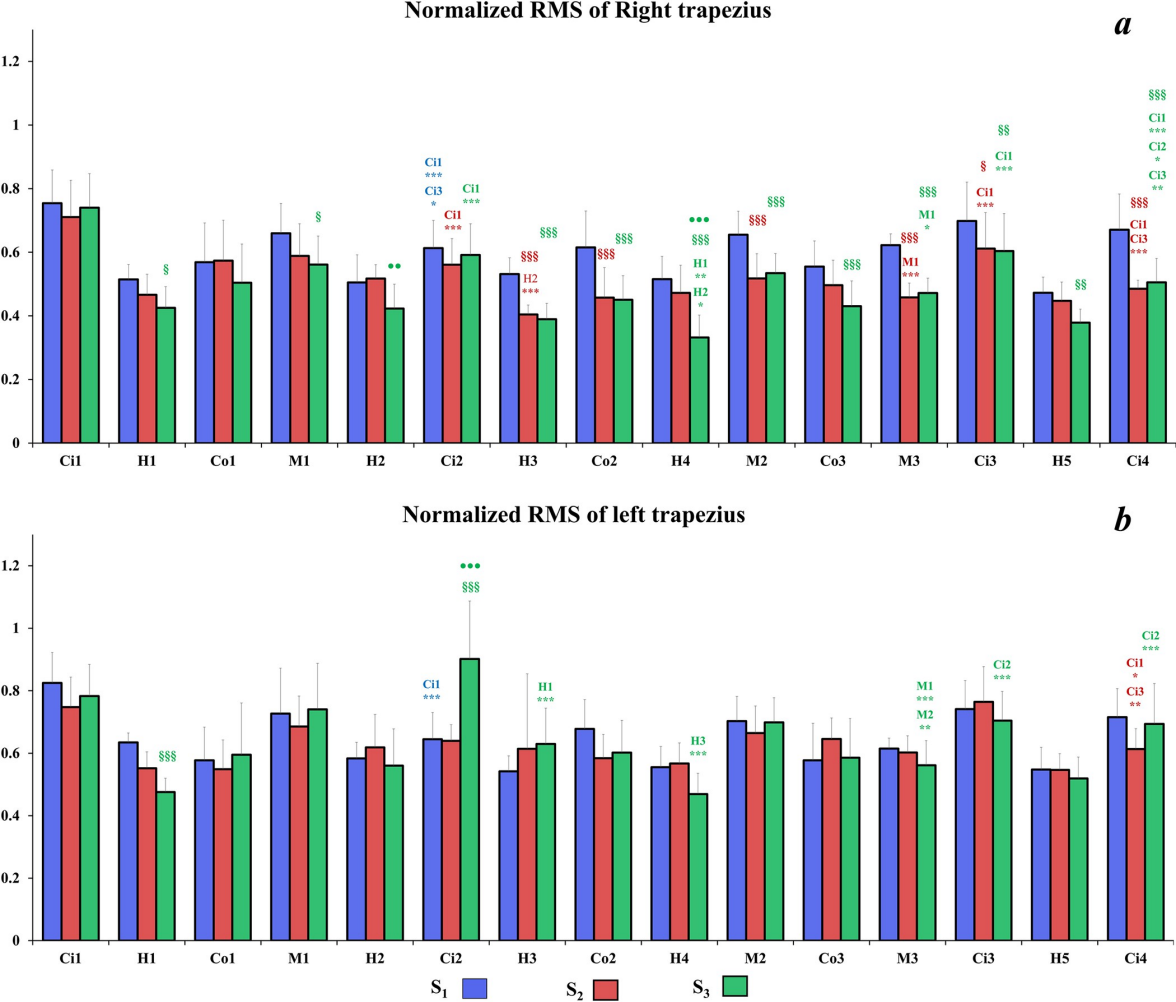
Mean of right (a) and left (b) TD normalized RMS values for each road sector for all seats. Significant differences between sectors of a same road type are symbolized by * (sectors concerned by the significant differences are mentioned above *); significant differences between S_1_ and other seats for a same road sector §; significant differences between S_2_ and S_3_ seats for a same road sector ●.

**Fig 7:**
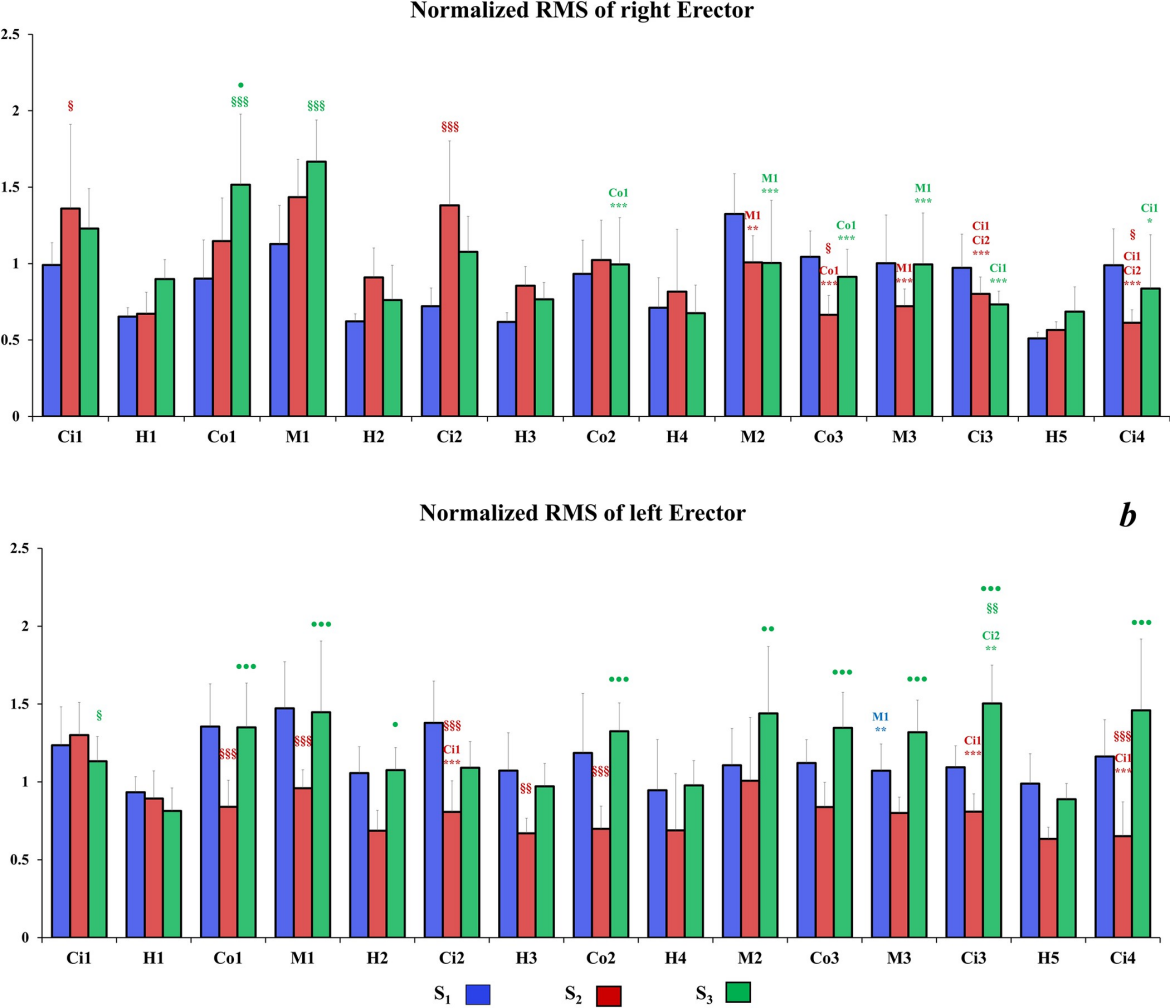
Mean of right (a) and left (b) ESL normalized RMS values for each road sector for all seats. Significant differences between sectors of a same road type are symbolized by * (sectors concerned by the significant differences are mentioned above *); significant differences between S_1_ and other seats for a same road sector §; significant differences between S_2_ and S_3_ seats for a same road sector ●.

**Fig 8:**
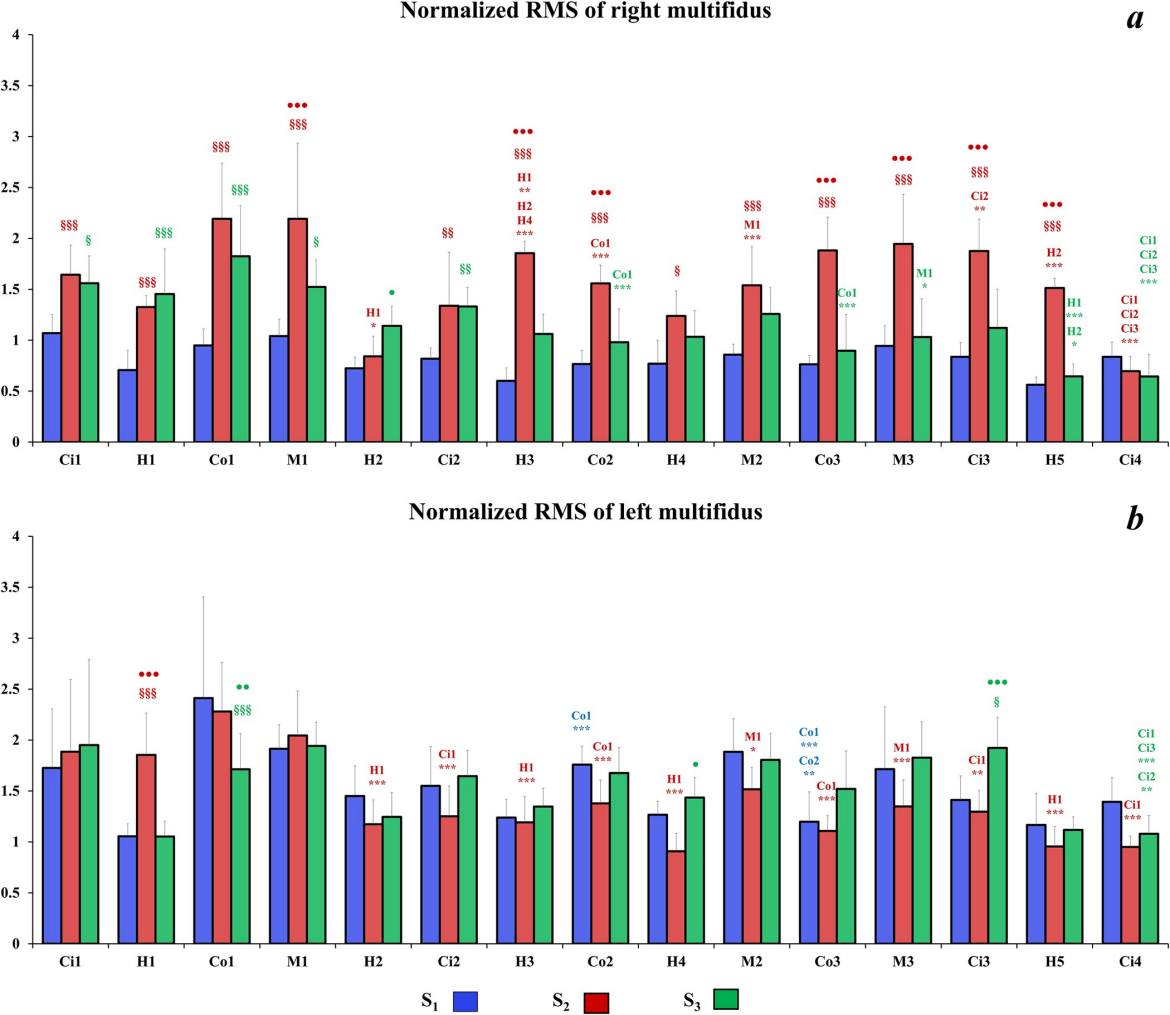
Mean of right (a) and left (b) MF normalized RMS values for each road sector for all seats. Significant differences between sectors of a same road type are symbolized by * (sectors concerned by the significant differences are mentioned above *); significant differences between S_1_ and other seats for a same road sector §; significant differences between S_2_ and S_3_ seats for a same road sector ●.

Regarding the description of the following results, we considered that a significant variation of normalized RMS corresponds to an adjustment of muscular activity. Concerning the **right TD** (**Fig 6A**), with both S_2_ and S_3_ seats, the RMS may decrease but also may increase during the driving session. However, with the S_1_ seat, the level of muscular activity remained stable throughout the driving task except for the Ci2 sector which presented lower normalized RMS compared to Ci1 (p<0.001) and Ci3 (p<0.05) sectors. Moreover, the normalized RMS values of right TD were higher with the S_1_ seat compared to the others in almost all road sectors. Interestingly, the normalized RMS was significantly lower for S_3_ seat compared to S_2_ seat in H2 and H4 sectors. Finally, at the end of the driving session, the level of muscular activity with S_2_ and S_3_ seats was significantly lower in City sectors (i.e., Ci3 and Ci4) than for S_1_ seat. For the **left TD** (**Fig 6B**), most of significant differences appeared for S_3_ for which normalized RMS level varied in several sectors (i.e., H3, H4; M3; Ci3 and Ci4). Moreover, some differences occurred for S_1_ and S_2_ respectively in Ci2 and Ci4 but for these seats the muscular activity remains quite stable. Similarly, to the right TD, muscular adjustments occurred for the **right ES** with S_2_ and S_3_ seats from Mountain 2 sector to the end of the driving session (**Fig 7A**). However, there was still no difference observed between sectors for the S_1_ seat throughout the driving task. Interestingly, at the beginning of the driving session, the normalized values of RMS were higher for S_3_ compared to the S_1_ in some sectors (i.e., Co1, M1 and Ci2). However, after the City 2 sector, the normalized RMS of right ES decreased with S_3_ and no significant difference was observed compared to the other seats. Concerning the **left ES** (**Fig 7B**), the muscular activity with S_2_ was lower than the other seat and did not reveals muscular adjustment throughout the driving session except for city sectors where normalized RMS values decreased compared to Ci1 sector. Moreover, normalized values of RMS were lower than for other seats in almost all sectors. Muscular activity remained stable for S_3_, and kept a higher level compared to other seats at the end of the driving session. Excepted for M3 where the normalized RMS values were lower than in M1 sector, there was still no significant compensation of muscular activity for S_1_. Concerning the **right MF** (**Fig 8A**), S_2_ induced higher normalized RMS values compared to the other seats. Furthermore, several significant adjustments of muscular activity occurred with this seat from the H3 sector onwards. The S_3_ seat induced less muscular activity compensation than S_2_, however a clear decrease of the normalized RMS is observed in the last road sectors compared to the first four ones. With S_1_, the normalized RMS values of right MF remained low and steady throughout the driving session. For the **left MF** (**Fig 8B**), there was almost no significant adjustment of the muscular activity for S_1_, except for country roads where normalized RMS values decreased significantly with time. With S_2_, the normalized RMS values of left MF were lower than on the right MF. Moreover, from H2 sector to the end of the driving task, the normalized RMS values decreased compared to the first sectors. The muscular activity of the left MF remained stable with S_3_ in all sectors except for Ci4 sector, which revealed lower normalized RMS value compared to previous city sectors.

## Discussion

A major issue for automotive companies is to improve well-being and reduce the discomfort of drivers. In this context, several studies have highlighted a deleterious effect of driving i.e. discomfort increase and NMF. In the present experiment, the impact of three different seats (one soft—S_1_, one firm—S_2_ and one innovative suspended seat—S_3_), on discomfort perception and muscular activity was assessed during three hours of real driving task. Our main results exhibit different seat-dependent neuromuscular profiles despite no significative differences in perceived discomfort. This highlights the necessity to use physiological parameters in order to objectively evaluate efficiency of new seat designed by automotive companies.

### Perceived discomfort

No significant differences were observed between seats. However, the whole-body discomfort starts to be significant later with the S_3_ seat (160-min) compared to S_2_ (80-min) and S_1_ (100-min). Regarding our previous results from a static simulator study where the whole-body discomfort increased significantly from 40 minutes for both S_1_ and S_2_ seats, the discomfort perception appears later in real driving conditions. This result is probably due to the dynamics aspect of real driving task as immobility (specific to the static simulator’s condition) is well known to be a negative factor for discomfort perception [7, 23]. Moreover, for almost all body-part areas the perceived local discomfort is lower with S_3_ compared to S_1_ and S_2_, and in particular for neck, buttocks and hind limbs. These positive results are probably due to the innovative functionality (suspended seat) of S_3_. However, the conception of S_3_ still needs improvements because it caused a high level of discomfort in the lower back area. The high level of perceived discomfort in arms was probably due to our driving instruction as we asked to participants to keep a maximum their hand on the steering wheel throughout the driving sessions.

### Endurance static tests

In order to characterize the effect of the three hours of driving session on physical capacity for each tested seat, EST were performed before and after the driving task. Such exercise consists in maintaining a load as long as possible and implies the recruitment of the main muscles adapted to the driving task but also synergetic and accessories muscles [37, 38]. Even if both TD muscles are mainly solicitated during the EST to maintain weight bar at pectoral level in a standing up position, back muscles like ES and MF also sustain the task. Interestingly, the results of EST demonstrate that endurance time significantly decrease following driving sessions with both S_1_ and S_2_ (mean decrease of -19 seconds), while there was no significant difference as before and after the driving task when S_3_ was used (mean decrease of -8 seconds). It is interesting to notice that EST was measured before and after long duration driving carried out on an identical itinerary and time of the day, with an automatic gearbox and respecting the driving rules. Consequently, we do assume that driving sessions were performed in very similar conditions. In addition, more than 3 hours separated the two EST tasks meaning that subjects had enough time to recover from the first test, regarding the fact that the level of muscle contractions is not continuous and not high during the driving task. Consequently, a difference between pre and post EST should be due to the effect of the driving task on muscle capacity and ergonomic features of the seats. This result is correlated to those of discomfort perception suggesting that the less discomfortable seat (S_3_) induces lower impairment of physical capacity.

### Muscular activity

**Fig 9:**
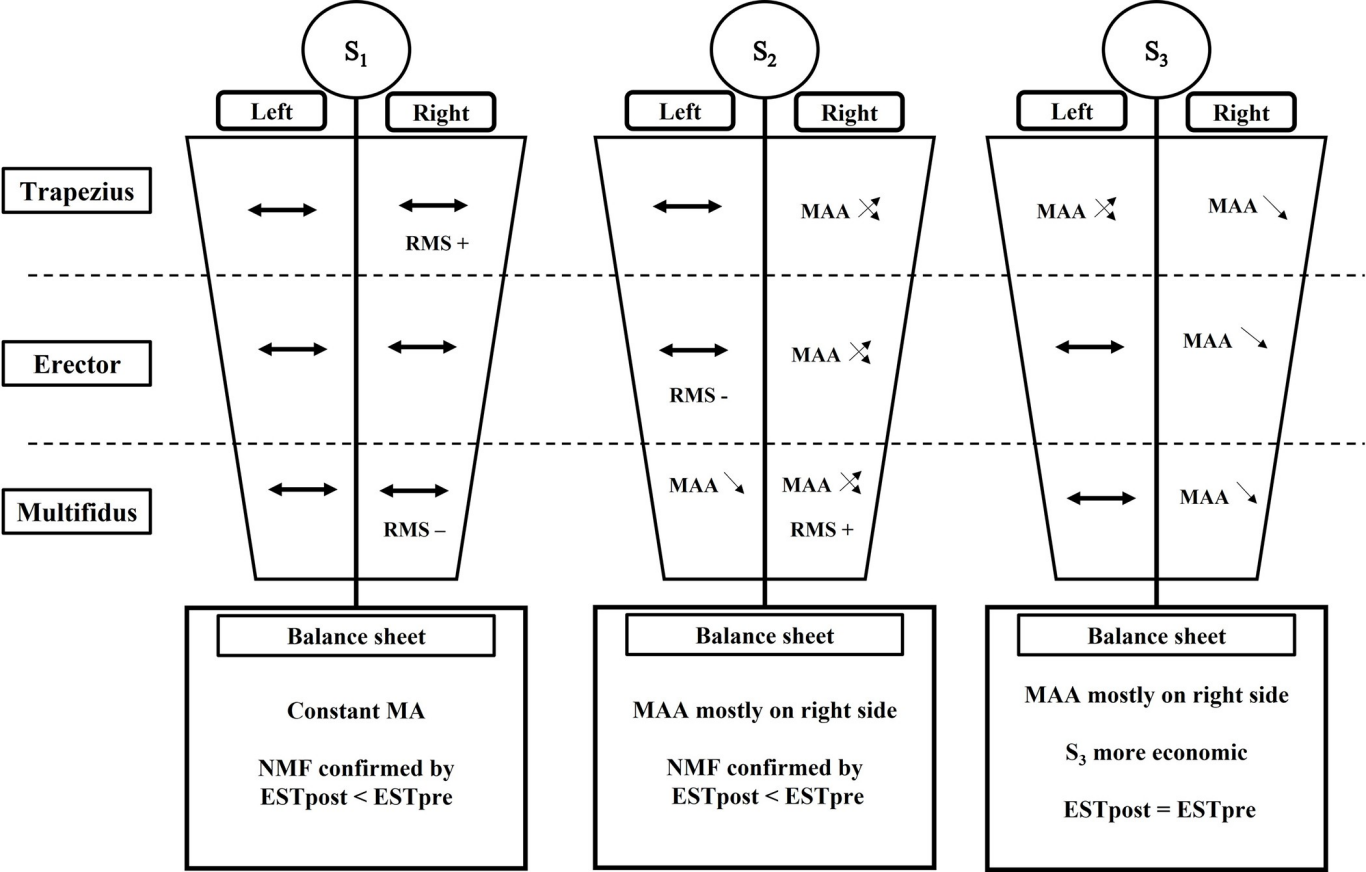
Schematic representation of muscular activity evolution of back muscles during the driving task for the three tested seats. With the S_1_ seat, the muscular activity remains mostly stable (horizontal bold arrow) for all muscles while there is muscular activity adjustments (MAA) for the two other seat. For a same seat, crossed arrows represent fluctuation of muscular activity during the driving task; down arrows represent diminution of muscular activity with driving time. Moreover, RMS+ and RMS- are indicated when for the same muscle a seat induces respectively higher or lower muscular activity compared to the other seats for most of road sectors.

General profiles of muscular activity during the driving task for each seat are summarized in **Fig 9**. Our results demonstrated that the first muscular adjustment mechanisms occurred after approximately 1h00 of driving for S_2_ and S_3_ seats. In comparison with our previous experiment on static simulator [34], these muscular adjustments appeared sooner in real driving conditions (approximately 90 minutes earlier). Real driving conditions could induce higher mental workload due to conscious risk related to traffic, which in turn could favor more anticipated muscle contractions.

Overall, our results show that with S_1_ there is almost no variation of muscular activity which mostly concerns S_2_ and S_3_. For the latter, the muscular activity adjustments occurred mainly on the right side for TD, ES and MF but with different profiles while, on left side, muscular adjustments concerns only MF with S_2_ and TD for S_3_. This result could be due to the direct link with the right leg which constantly interacts with the pedals. Consequently, to maintain posture and driving task in these conditions, muscle tone might be more elevated on the right side than on the left side. Another possibility could be, as most of the drivers were right-handed, that they probably use right hand’s muscles more than their left to turn the steering wheel.

In more details, regarding the muscular activity of trapezius, S_3_ is the only seat with which fluctuations occurred on left TD whereas it remained stable for S_1_ and S_2_. Moreover, for right TD, muscular activity was significantly lower for S_3_ compared to S_2_ in H2 and H4. These results suggest a greater muscular relaxation of TD muscles for S_3_ on the highway sectors. This assumption is confirmed by the fact that in the following sectors the muscular activity increases again for S_3_ and then decreases again in H5. Our results are consistent with those for Kia et al., [39] who demonstrated that dynamic seats tend to reduce WBV and muscular activity of left trapezius (~20–40%) and left splenius capitis (~10–30%) when road vibration profiles are reproduced on a motion platform. Regarding muscular activity of ES muscles, with S_3_, the normalized RMS values of right ES was higher at the beginning of the driving session compared to the other seat but following the Ci2 sector it returned to the same level. Moreover, muscular activity of left ES remains stable for all seats but with S_2_ the level of normalized RMS was lower compared to S_1_ and S_3_. Such particularity is probably due to the firmness of S_2_ which provides higher support for buttock and lower back, reducing the muscular contribution of left ES to maintain driving posture. Interestingly, with S_2_ the muscular activity of right MF was higher while it was lower for left MF compared to the two other seats. This result is consistent with those from our previous experiment on static simulator. Our main assumption is that, thanks to its firmness, S_2_ provides a better support to the drivers’ back, leading to less muscular activity adjustment on the left side. Consequently, muscular contractions occurred mainly on the right side to compensate the action of the right leg on the pedals. Due to its firmness and lower vibration absorption, such compensation mechanisms are more important for S_2_ than for S_3_. This results in a significant decrease of ESTpost compared to ESTpre and earlier perceived discomfort with S_2_. With S_1_, the global level of muscular activity remains stable for all sectors of the same road type for all recorded muscles. This might be interpreted as an absence of muscular adjustment despite impairment in muscles capacity to produce a force. This in turn is confirmed by the lower endurance time during ESTpost compared to ESTpre with this seat. This muscle impairment could be a consequence of higher normalized RMS values of right TD with S_1_ compared to the other seats in almost all road sectors. These higher values are coherent with results obtained in S_2_ and S_3_ indicating that right side muscular activity remained elevated to compensate right leg activity. However, the steady aspect of muscular activity observed with S_1_ could be related to a decreased capacity to react to the physical stimuli induced by driving, due to the softer features of the foam allowing the body to sink further into the seat. Consequently, the muscles involved in maintaining the driving posture have a sustained contraction without any compensation strategy between muscles. This would account for the higher level of neuromuscular impairment at the end of the driving session (decreased endurance time).

Our results demonstrated that S_3_ could be considered as the most suitable seat to delay perceived discomfort and reduce neuromuscular impairment. The mechanical spring directly incorporated into the seat backrest allows the absorption of more vibrations inducing slight vertical movements directly related to road asperity. Although mostly used for buses and semi-trucks, dynamic seats are recognized as an efficient means of reducing WBV, spinal shrinkage and discomfort by allowing relaxation period of neck and back extensor muscles [39–43]. Thus, by generating more movements in several sectors, S_3_ favors a better muscle compensation strategy, which finally results in an absence of significant difference of ESTpost compared to ESTpre and delayed perceived discomfort.

### Link with pressure parameters

In the same experimental protocol, data of contact pressure (CP) and contact surface (CS) were also synchronously recorded for each seat. The corresponding results have already been published by Lantoine et al., [35]. It is interesting to notice that, firstly, CP and CS profiles of backrest were identical for the three seats tested. Moreover, right upper back area revealed lower CP and CS compared to the other back areas for each seat (i.e., left upper back; right and left lower back). This repartition profile is associated with high level of muscular activity adjustments for S_2_ and S_3_ (right TD and ES) while they remained stable for S_1_. Concerning the cushion, S_1_ showed a uniform CP repartition between thighs and buttocks which is certainly due to more pronounced body sinking. This result could be related to the stable back muscular activity caused by S_1_. Moreover, even if S_3_ is identified as the softest cushion, like S_2,_ it induced a non-uniform CP repartition between thighs and buttocks throughout the driving sessions. The suspended system included in S_3_ and the firmness of S_2_ seem to result in better postural support with higher thighs CP allowing more muscular adjustments. These interdependent effects between the trunk and the lower limbs confirmed the lumbo-pelvic interaction in driving posture depending on seat type [29].

### Methodological considerations

Several methodological considerations should be taken into account regarding the present study. First, contrary to our previous experiments performed on a static simulator [34, 44], we choose here to realize real driving sessions. This ecological approach is justified by our objective to investigate drivers’ muscular adaptation and discomfort perception related to different seats in normal conditions. However, even if the itinerary was identical, variation could occur between driving sessions due to traffic, climatic conditions, and roadworks. Otherwise, before each driving session, participants were asked to adjust their seat freely to be as comfortable as possible but were warned that they would not be able to modify their settings during the driving task. Thus, from one experimental handover to another, there might be variability concerning the seat settings for a same participant [45]. Moreover, because of their own anthropometric features and personal preferences each participant did not have the same seat settings. The consequent variability of forward/backward adjustment, backrest inclination and body-segments angles may have some incidences on muscular activity and perceived discomfort. Furthermore, to overcome arms’ unactive position variability during the driving task, all participants were instructed to maintain their hands on the steering-wheel without the use of armrests throughout the driving task. This does not accurately reflect a natural position and with time, this constraint possibly amplified their perceived discomfort for the upper limbs and modified their way of driving. Accelerometric measurements were not performed, however it could be pertinent to record the vibration profiles of seat rails, seat foam, and drivers’ bodies to assess in detail the mechanical transfer function according of each seat for the complete itinerary of our experiment. Another limitation of the study concerns EMG analysis. First of all, we choose to not use frequency analysis as it remains controversial. Indeed, the EMG power spectrum could be related to changes in motor unit recruitment as well as changes in firing frequency with no capacity to distinguish the participation of both recruitments. Moreover, frequency analysis depends also on various parameters independent of muscle recruitment such as muscle length and angle, skin conductance (acting as low-pass filter), electrodes placement. It seems, that frequency analysis remains suitable and promising in cases of isometric contractions [46], however in dynamic conditions it appeared that according to the chosen size of FFT windows, MF exhibits remarkable changes [47, 48]. Moreover, Farfan et al. [46] have demonstrated that the best processing technique in dynamic conditions is the EMG-RMS. In our conditions where contractions are intermittent and variable, contrary to EMG-RMS analysis, frequency analysis would be influenced by true physiological events as well as methodological or biomechanical artifacts. For these reasons, EMG-RMS remained a more appropriated methods to analyze neural strategy involved in long-duration driver. Finally, regarding normalization procedure it is admitted that the best way to normalize RMS signals is with respect to RMS values recorded during maximal voluntary contraction. However, in our conditions, precise measurement of an MVC produced by our selected muscles is quite difficult and even impossible with a clear accuracy.

## Conclusion

This second experiment, in line with the first, considers our ability to objectify neuromuscular fatigue during long duration driving condition. Once again, our results highlighted different neuromuscular fatigue profiles according to each seat. The firmer structure of S_2_ represents a more suitable support for the back leading to neural compensation strategies. The prototyped seat (S_3_) seems to be promising as we found also compensation strategy as for the S_2_ but with less muscular activity leading to a better tolerance to neuromuscular fatigue as confirmed by the absence of significant difference in endurance time test between ESTpre and ESTpost. Finally, although the subject could perceive a softer seat such as S_1_ as comfortable, this impression does not reflect the real body solicitation. Indeed, with S_1_, the reduced back support leads to an absence of compensation strategy, which, in turn, results in a greater reduction in endurance time. Despite the absence of significant difference in this particular result between S_1_ and S_2_, we can notice a slight tendency of a greater reduction in S_1_ compared to S_2_. Contrary to several studies, we propose here a standardized and robust methodology to assess the efficiency of several car seats from automotive companies with similar experimental protocols. So considered, it seems that we now have the capability and skills to distinguish the different fatigue profiles generated with different types of seats during long duration driving. It will be interesting to pursue our research by studying the specific profiles of passengers during long duration driving and by evaluating the behavioral differences between driver and passenger for a same seat.
